# Aquabis(benzoato-κ*O*)bis­(1*H*-imidazole-κ*N* ^3^)copper(II) monohydrate

**DOI:** 10.1107/S1600536809007600

**Published:** 2009-03-06

**Authors:** Bin-Bin Liu, Ting Weng, Hong-Zhen Xie

**Affiliations:** a State Key Laboratory Base of Novel Functional Materials and Preparation Science, Faculty of Materials Science and Chemical Engineering, Institute of Solid Materials Chemistry, Ningbo University, Ningbo, Zhejiang, 315211, People’s Republic of China

## Abstract

In the title compound, [Cu(C_7_H_5_O_2_)_2_(C_3_H_4_N_2_)_2_(H_2_O)]·H_2_O, the Cu^II^ atom is in a distorted square-pyramidal environment. The mol­ecules are assembled into double chains extending along [010] by N—H⋯O hydrogen bonds. These double chains are linked by O—H⋯O hydrogen bonds, forming layers parallel to (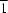
02); the layers are linked into a three-dimensional network by van der Waals inter­actions.

## Related literature

For general background, see: Escriva *et al.* (1996 ▶); Mu *et al.* (2002 ▶); Tian & Chen (2001 ▶). For related structures, see: Wang *et al.* (1999 ▶); Addison *et al.* (1984 ▶).
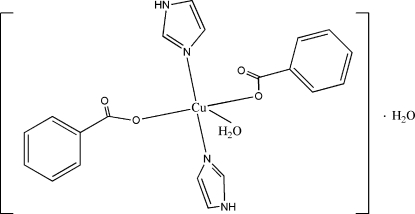


## Experimental

### 

#### Crystal data

[Cu(C_7_H_5_O_2_)_2_(C_3_H_4_N_2_)_2_(H_2_O)]·H_2_O
*M* *_r_* = 477.96
Monoclinic, 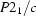

*a* = 18.366 (4) Å
*b* = 6.0076 (12) Å
*c* = 23.123 (9) Å
β = 122.64 (2)°
*V* = 2148.4 (12) Å^3^
*Z* = 4
Mo *K*α radiation
μ = 1.06 mm^−1^
*T* = 293 K
0.60 × 0.27 × 0.26 mm

#### Data collection

Rigaku R-AXIS RAPID diffractometer
Absorption correction: multi-scan (*ABSCOR*; Higashi, 1995 ▶) *T*_min_ = 0.710, *T*_max_ = 0.750
19722 measured reflections
4867 independent reflections
3715 reflections with *I* > 2σ(*I*)
*R*_int_ = 0.034

#### Refinement

*R*[*F* ^2^ > 2σ(*F* ^2^)] = 0.033
*wR*(*F* ^2^) = 0.120
*S* = 1.15
4867 reflections
281 parameters
H-atom parameters constrained
Δρ_max_ = 0.66 e Å^−3^
Δρ_min_ = −0.57 e Å^−3^

### 

Data collection: *RAPID-AUTO* (Rigaku, 1998 ▶); cell refinement: *RAPID-AUTO*; data reduction: *CrystalStructure* (Rigaku/MSC, 2004 ▶); program(s) used to solve structure: *SHELXS97* (Sheldrick, 2008 ▶); program(s) used to refine structure: *SHELXL97* (Sheldrick, 2008 ▶); molecular graphics: *ORTEPII* (Johnson, 1976 ▶); software used to prepare material for publication: *SHELXL97*.

## Supplementary Material

Crystal structure: contains datablocks ptcla, I. DOI: 10.1107/S1600536809007600/ng2555sup1.cif

Structure factors: contains datablocks I. DOI: 10.1107/S1600536809007600/ng2555Isup2.hkl

Additional supplementary materials:  crystallographic information; 3D view; checkCIF report

## Figures and Tables

**Table 1:** Selected geometric parameters (Å, °)

Cu—O3	1.9765 (18)
Cu—O1	1.9814 (19)
Cu—N1	1.982 (2)
Cu—N3	1.984 (2)
Cu—O5	2.297 (2)

**Table 2:** Hydrogen-bond geometry (Å, °)

*D*—H⋯*A*	*D*—H	H⋯*A*	*D*⋯*A*	*D*—H⋯*A*
N2—H2*A*⋯O6^i^	0.86	1.88	2.729 (4)	172
N4—H19*A*⋯O1^ii^	0.86	2.03	2.886 (3)	177
O5—H51⋯O2^iii^	0.99	1.96	2.730 (3)	133
O5—H52⋯O4^iii^	0.97	2.06	2.873 (4)	141
O6—H61⋯O4^iii^	0.85	1.94	2.729 (4)	155
O6—H62⋯O3	0.86	1.99	2.827 (3)	165

Symmetry codes: (i) 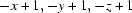; (ii) 
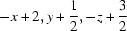
; (iii) 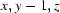.

**Fig. 1.**
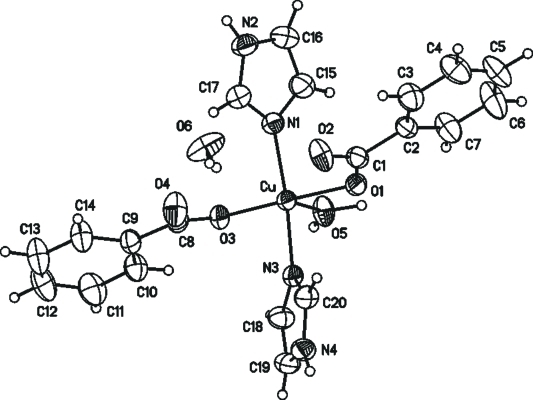
ORTEP view of the title compound. The dispalcement ellipsoids are drawn at 45% probability level.

**Fig. 2.**
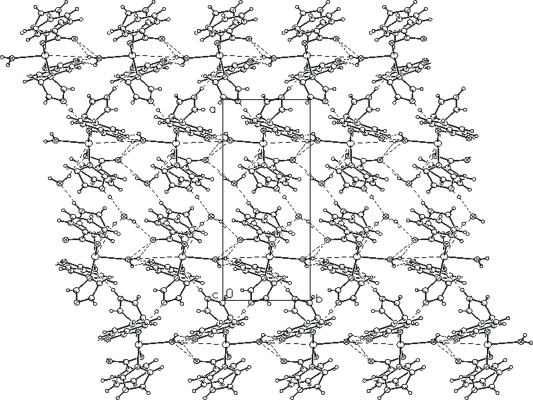
The double chains of the title complex pallel to [010].

## supplementary crystallographic information

### Comment

The copper(II) complexes with carboxylic acid exist extensively and play an important role in a vast range of chemistry and organisums (Escriva, *et al.*, 1996; Tian & Chen, 2001). At the same time, some copper(II) complexes with imidazole ligand show special properties, such as optical properties, magnetic properties and activity of superoxide dismutase, Which have potential applications in material and medicine industry(Mu, *et al.*, 2002). However, investigation of combination copper(II), carboxylic acid and imidazole to design monomeric copper(II) complexes is quite limited. Herein, we report the synthesis, crystal structure of a novel complex [Cu(H_2_O)(C_3_H_4_N_2_)_2_ (C_6_H_5_COO)_2_].H_2_O.

The asymmetric unit of the title compound consists of one Cu(II) ion, two imidazole molecules, two benzoate anions, one aqua ligand and one lattice H_2_O molecule, As illustrated in Fig. 1. The copper atom is involved in a CuN_2_O_3_ chromophore and lies in a distorted square-pyramidal environment. The equatorial positions are occupied by two oxygen atoms from two different benzoate anions and two nitrogen atoms from two different imidazole molecules. The two nitrogen atoms from imidazole ligands and two carboxylate oxygen atoms are in a *trans* position, as observed in other compounds of copper(II) (Wang, *et al.*, 1999), and the axial position is occupied by O5 atoms. The bond lengths of Cu—N1 and Cu—N3 fall in the range of 1.982 (2) and 1.984 (2) Å, and the Cu—O1 and Cu—O3 bond distances are equal to 1.977 (2) and 1.982 (2) Å, the axial Cu—O5 bond distance is 2.297 (2) Å. The τ index about the central Cu atom is 0.156 Å, suggesting that the square pyramidal coordination geometry is slightly distorted (Addison *et al.*, 1984). The bond length of Cu—O2 and Cu—O4 are 3.034 (3) and 3.040 (3) Å, which is longer than normal bond length of Cu—O, indicating there is no interaction between Cu—O2 and Cu—O4. There are two different benzoate anions in the compound. The plane of benzene ring and carboxylate exhibit nearly perfect coplanarity in the benzoate anion containing C1, but for another benzoate anion, the dihedral angle between benzene ring and carboxylate plane is 16.6 (6)°. The two imidazole ligands are not distortion in the compound and the dihedral angle between two imidazole rings is 67.5 (1)°C. The complex molecules are assembled into one dimension chains extending along the [010] direction through hydrogen bonds between aqua ligand and uncoordinated carboxylate oxygen atoms (O5—H51···O2, O5—H52···O4). Then the one dimension chains generate double chains through hydrogen bonds by nitrogen atom of imidazole provide H19A to carboxylate oxygen atom (O1), the double chains are assembled further into two-dimensional layer parallel to (-1 0 2) by hydrogen bonded of N2—H2A···O6 and O6—H62···O3, the two dimension layers array alternately to generate three dimension network by Van der Waals interactions.

### Experimental

After CuCl_2_.2H_2_O (0.1702 g, 1.001 mmol), benzoic acid (0.1221 g, 1.000 mmol) and imidazole (0.1354 g, 1.002 mmol) were completely dissolved in 20 ml mixed solvent of H_2_O and CH_3_CH_2_OH (V_w_:V_e_ = 1:1). Then 1.2 ml (1.0 *M*) NaOH was dropwise added and the resulting dark blue suspension (pH = 8.0) was subsequently allowed to stir for 1 h. After the suspension was filtrated, the filtrate was allowed to stand at room temperature. The block-like crystals were obtained twenty days later. IR spectroscopic analysis (KBr, υ/cm^-1^): 3424(w), 1600(*m*), 1560(*m*), 1543(w), 1387(*m*), 1068(*m*), 717(*m*).

### Refinement

H atoms bonded to C atoms were palced in geometrically calculated positionand were refined using a riding model, with *U*_iso_(H) = 1.5 *U*_eq_(C). H atoms attached to O atoms were found in a difference Fourier synthesisand were refined using a riding model, with the O—H distances fixed as initially found and with *U*_iso_(H) values set at 1.5 *U*eq(O).

### Crystal data

**Table:**

[Cu(C_7_H_5_O_2_)_2_(C_3_H_4_N_2_)_2_(H_2_O)]·H_2_O	*F*(000) = 988
*M**_r_* = 477.96	*D*_x_ = 1.478 Mg m^−^^3^
Monoclinic, *P*2_1_/*c*	Mo *K*α radiation, λ = 0.71073 Å
Hall symbol: -P 2ybc	Cell parameters from 19722 reflections
*a* = 18.366 (4) Å	θ = 3.0–27.4°
*b* = 6.0076 (12) Å	µ = 1.06 mm^−^^1^
*c* = 23.123 (9) Å	*T* = 293 K
β = 122.64 (2)°	Block, purple
*V* = 2148.4 (12) Å^3^	0.60 × 0.27 × 0.26 mm
*Z* = 4	

### Data collection

**Table:**

Rigaku R-AXIS RAPID diffractometer	4867 independent reflections
Radiation source: fine-focus sealed tube	3715 reflections with *I* > 2σ(*I*)
graphite	*R*_int_ = 0.034
Detector resolution: 0 pixels mm^-1^	θ_max_ = 27.4°, θ_min_ = 3.0°
ω scans	*h* = −23→23
Absorption correction: multi-scan (*ABSCOR*; Higashi, 1995)	*k* = −7→7
*T*_min_ = 0.710, *T*_max_ = 0.750	*l* = −29→29
19722 measured reflections	

### Refinement

**Table:**

Refinement on *F*^2^	Secondary atom site location: difference Fourier map
Least-squares matrix: full	Hydrogen site location: inferred from neighbouring sites
*R*[*F*^2^ > 2σ(*F*^2^)] = 0.033	H-atom parameters constrained
*wR*(*F*^2^) = 0.120	*w* = 1/[σ^2^(*F*_o_^2^) + (0.0588*P*)^2^ + 0.849*P*] where *P* = (*F*_o_^2^ + 2*F*_c_^2^)/3
*S* = 1.15	(Δ/σ)_max_ = 0.002
4867 reflections	Δρ_max_ = 0.66 e Å^−^^3^
281 parameters	Δρ_min_ = −0.57 e Å^−^^3^
0 restraints	Extinction correction: *SHELXL*, Fc^*^=kFc[1+0.001xFc^2^λ^3^/sin(2θ)]^-1/4^
Primary atom site location: structure-invariant direct methods	Extinction coefficient: 0.0058 (7)

### Special details

**Table:**

Geometry. All e.s.d.'s (except the e.s.d. in the dihedral angle between two l.s. planes) are estimated using the full covariance matrix. The cell e.s.d.'s are taken into account individually in the estimation of e.s.d.'s in distances, angles and torsion angles; correlations between e.s.d.'s in cell parameters are only used when they are defined by crystal symmetry. An approximate (isotropic) treatment of cell e.s.d.'s is used for estimating e.s.d.'s involving l.s. planes.
Refinement. Refinement of *F*^2^ against ALL reflections. The weighted *R*-factor *wR* and goodness of fit *S* are based on *F*^2^, conventional *R*-factors *R* are based on *F*, with *F* set to zero for negative *F*^2^. The threshold expression of *F*^2^ > σ(*F*^2^) is used only for calculating *R*-factors(gt) *etc*. and is not relevant to the choice of reflections for refinement. *R*-factors based on *F*^2^ are statistically about twice as large as those based on *F*, and *R*- factors based on ALL data will be even larger.

### Fractional atomic coordinates and isotropic or equivalent isotropic displacement parameters (Å^2^)

**Table:**

	*x*	*y*	*z*	*U*_iso_*/*U*_eq_	
Cu	0.781397 (17)	0.46304 (5)	0.649481 (14)	0.03036 (13)	
O1	0.84502 (12)	0.4688 (3)	0.60237 (9)	0.0404 (4)	
O2	0.80700 (15)	0.8199 (3)	0.56883 (12)	0.0562 (6)	
C1	0.83217 (16)	0.6360 (5)	0.56317 (13)	0.0373 (6)	
C2	0.84906 (16)	0.5985 (4)	0.50741 (13)	0.0356 (5)	
C3	0.8361 (2)	0.7703 (5)	0.46303 (16)	0.0517 (7)	
H3A	0.8166	0.9077	0.4679	0.062*	
C4	0.8520 (2)	0.7397 (6)	0.41151 (17)	0.0644 (9)	
H4A	0.8424	0.8560	0.3816	0.077*	
C5	0.8814 (3)	0.5408 (6)	0.40419 (18)	0.0669 (10)	
H5A	0.8936	0.5224	0.3703	0.080*	
C6	0.8931 (3)	0.3686 (6)	0.4468 (2)	0.0767 (11)	
H6A	0.9124	0.2318	0.4413	0.092*	
C7	0.8765 (2)	0.3957 (5)	0.49800 (17)	0.0567 (8)	
H7A	0.8840	0.2764	0.5263	0.068*	
O3	0.71958 (11)	0.4369 (3)	0.69750 (9)	0.0362 (4)	
O4	0.69831 (15)	0.8000 (3)	0.69427 (12)	0.0571 (6)	
C8	0.69452 (15)	0.6102 (4)	0.71336 (13)	0.0359 (5)	
C9	0.65960 (17)	0.5768 (4)	0.75849 (14)	0.0382 (6)	
C10	0.67548 (19)	0.3796 (5)	0.79480 (15)	0.0460 (7)	
H10A	0.7046	0.2638	0.7890	0.055*	
C11	0.6480 (2)	0.3556 (6)	0.83958 (18)	0.0622 (9)	
H11A	0.6608	0.2261	0.8654	0.075*	
C12	0.6016 (3)	0.5241 (6)	0.8460 (2)	0.0694 (10)	
H12A	0.5825	0.5069	0.8757	0.083*	
C13	0.5835 (3)	0.7162 (6)	0.8088 (2)	0.0687 (10)	
H13A	0.5511	0.8277	0.8125	0.082*	
C14	0.6135 (2)	0.7444 (5)	0.76576 (18)	0.0537 (8)	
H14A	0.6025	0.8768	0.7416	0.064*	
N1	0.67162 (14)	0.4759 (4)	0.55835 (11)	0.0384 (5)	
N2	0.55699 (14)	0.6160 (5)	0.46720 (12)	0.0502 (6)	
H2A	0.5156	0.7060	0.4411	0.060*	
N3	0.88911 (13)	0.5247 (3)	0.73940 (11)	0.0335 (5)	
N4	1.00928 (13)	0.6723 (4)	0.82320 (12)	0.0433 (5)	
H19A	1.0535	0.7585	0.8447	0.052*	
C15	0.64338 (19)	0.3320 (5)	0.50435 (15)	0.0524 (7)	
H15A	0.6690	0.1965	0.5061	0.063*	
C16	0.5725 (2)	0.4178 (6)	0.44808 (16)	0.0598 (9)	
H16A	0.5406	0.3532	0.4047	0.072*	
C17	0.61699 (17)	0.6456 (5)	0.53335 (14)	0.0456 (7)	
H17A	0.6202	0.7694	0.5588	0.055*	
C18	0.91443 (17)	0.4149 (5)	0.79964 (14)	0.0431 (6)	
H18A	0.8851	0.2965	0.8040	0.052*	
C19	0.98817 (19)	0.5051 (5)	0.85127 (14)	0.0465 (7)	
H4B	1.0167	0.4654	0.8937	0.056*	
C20	0.94874 (16)	0.6780 (5)	0.75607 (14)	0.0396 (6)	
H20A	0.9486	0.7772	0.7251	0.047*	
O5	0.79582 (13)	0.0829 (3)	0.66003 (10)	0.0453 (5)	
H51	0.8286	0.0378	0.6393	0.068*	
H52	0.7906	−0.0310	0.6870	0.068*	
H62	0.6186	0.2392	0.6401	0.068*	
O6	0.58185 (13)	0.1323 (4)	0.62429 (13)	0.0706 (7)	
H61	0.6070	0.0195	0.6493	0.106*	

### Atomic displacement parameters (Å^2^)

**Table:**

	*U*^11^	*U*^22^	*U*^33^	*U*^12^	*U*^13^	*U*^23^
Cu	0.02862 (18)	0.03451 (19)	0.02904 (18)	0.00121 (11)	0.01627 (13)	0.00204 (11)
O1	0.0354 (9)	0.0570 (11)	0.0337 (10)	0.0072 (8)	0.0218 (8)	0.0102 (8)
O2	0.0826 (15)	0.0443 (11)	0.0648 (14)	−0.0019 (11)	0.0549 (13)	−0.0088 (10)
C1	0.0347 (12)	0.0450 (15)	0.0330 (13)	−0.0058 (12)	0.0188 (11)	−0.0043 (11)
C2	0.0368 (12)	0.0396 (13)	0.0313 (12)	−0.0038 (11)	0.0190 (11)	−0.0019 (10)
C3	0.0671 (19)	0.0465 (16)	0.0508 (18)	0.0065 (14)	0.0379 (16)	0.0094 (13)
C4	0.089 (3)	0.067 (2)	0.0488 (19)	−0.0036 (19)	0.0452 (19)	0.0107 (16)
C5	0.097 (3)	0.075 (2)	0.052 (2)	−0.012 (2)	0.056 (2)	−0.0123 (17)
C6	0.125 (3)	0.058 (2)	0.082 (3)	0.009 (2)	0.079 (3)	−0.006 (2)
C7	0.087 (2)	0.0435 (15)	0.0544 (18)	0.0083 (16)	0.0480 (18)	0.0044 (14)
O3	0.0372 (9)	0.0384 (9)	0.0406 (10)	−0.0002 (8)	0.0261 (8)	−0.0017 (8)
O4	0.0769 (14)	0.0407 (11)	0.0823 (16)	0.0119 (10)	0.0615 (14)	0.0192 (11)
C8	0.0322 (12)	0.0393 (13)	0.0394 (14)	0.0018 (11)	0.0213 (11)	0.0040 (11)
C9	0.0398 (13)	0.0412 (14)	0.0409 (14)	−0.0016 (11)	0.0265 (12)	−0.0022 (11)
C10	0.0563 (16)	0.0410 (14)	0.0529 (17)	0.0020 (14)	0.0374 (14)	0.0054 (13)
C11	0.084 (2)	0.0556 (19)	0.068 (2)	−0.0025 (18)	0.055 (2)	0.0101 (17)
C12	0.095 (3)	0.075 (2)	0.077 (3)	−0.009 (2)	0.072 (2)	−0.004 (2)
C13	0.087 (3)	0.066 (2)	0.087 (3)	0.0076 (19)	0.070 (2)	−0.004 (2)
C14	0.070 (2)	0.0461 (16)	0.069 (2)	0.0076 (14)	0.0527 (18)	0.0039 (14)
N1	0.0322 (11)	0.0478 (13)	0.0335 (11)	0.0006 (9)	0.0166 (9)	0.0012 (10)
N2	0.0379 (12)	0.0646 (16)	0.0378 (13)	0.0079 (12)	0.0137 (10)	0.0108 (12)
N3	0.0315 (10)	0.0380 (11)	0.0330 (11)	0.0014 (9)	0.0187 (9)	0.0016 (9)
N4	0.0323 (10)	0.0548 (14)	0.0382 (12)	−0.0128 (10)	0.0160 (10)	−0.0099 (11)
C15	0.0520 (16)	0.0533 (17)	0.0444 (16)	0.0060 (14)	0.0210 (14)	−0.0063 (14)
C16	0.0525 (17)	0.075 (2)	0.0369 (16)	0.0024 (17)	0.0143 (14)	−0.0081 (15)
C17	0.0404 (14)	0.0504 (16)	0.0416 (15)	0.0042 (13)	0.0192 (12)	0.0025 (13)
C18	0.0429 (14)	0.0490 (16)	0.0355 (14)	−0.0060 (13)	0.0198 (12)	0.0066 (12)
C19	0.0418 (14)	0.0629 (18)	0.0302 (13)	0.0002 (13)	0.0163 (12)	0.0032 (12)
C20	0.0354 (12)	0.0432 (14)	0.0404 (14)	−0.0075 (11)	0.0207 (11)	0.0017 (12)
O5	0.0619 (12)	0.0313 (9)	0.0619 (13)	0.0006 (8)	0.0460 (11)	−0.0003 (8)
O6	0.0401 (11)	0.0495 (13)	0.0839 (17)	−0.0027 (10)	0.0083 (11)	0.0156 (12)

### Geometric parameters (Å, °)

**Table:**

Cu—O3	1.9765 (18)	C12—C13	1.369 (5)
Cu—O1	1.9814 (19)	C12—H12A	0.9300
Cu—N1	1.982 (2)	C13—C14	1.383 (4)
Cu—N3	1.984 (2)	C13—H13A	0.9300
Cu—O5	2.297 (2)	C14—H14A	0.9300
O1—C1	1.286 (3)	N1—C17	1.324 (4)
O2—C1	1.232 (3)	N1—C15	1.370 (4)
C1—C2	1.497 (4)	N2—C17	1.329 (4)
C2—C7	1.380 (4)	N2—C16	1.353 (4)
C2—C3	1.383 (4)	N2—H2A	0.8600
C3—C4	1.382 (4)	N3—C20	1.319 (3)
C3—H3A	0.9300	N3—C18	1.376 (3)
C4—C5	1.359 (5)	N4—C20	1.336 (3)
C4—H4A	0.9300	N4—C19	1.361 (4)
C5—C6	1.363 (5)	N4—H19A	0.8600
C5—H5A	0.9300	C15—C16	1.351 (4)
C6—C7	1.383 (5)	C15—H15A	0.9300
C6—H6A	0.9300	C16—H16A	0.9300
C7—H7A	0.9300	C17—H17A	0.9300
O3—C8	1.270 (3)	C18—C19	1.345 (4)
O4—C8	1.238 (3)	C18—H18A	0.9300
C8—C9	1.504 (4)	C19—H4B	0.8600
C9—C14	1.383 (4)	C20—H20A	0.9300
C9—C10	1.389 (4)	O5—H51	0.9887
C10—C11	1.382 (4)	O5—H52	0.9646
C10—H10A	0.9300	O6—H62	0.8577
C11—C12	1.383 (5)	O6—H61	0.8477
C11—H11A	0.9300		
			
O3—Cu—O1	176.36 (8)	C12—C11—H11A	120.0
O3—Cu—N1	92.13 (9)	C13—C12—C11	120.2 (3)
O1—Cu—N1	88.75 (9)	C13—C12—H12A	119.9
O3—Cu—N3	88.64 (9)	C11—C12—H12A	119.9
O1—Cu—N3	91.29 (9)	C12—C13—C14	120.0 (3)
N1—Cu—N3	167.00 (9)	C12—C13—H13A	120.0
O3—Cu—O5	85.96 (7)	C14—C13—H13A	120.0
O1—Cu—O5	90.42 (7)	C13—C14—C9	120.4 (3)
N1—Cu—O5	98.31 (9)	C13—C14—H14A	119.8
N3—Cu—O5	94.69 (8)	C9—C14—H14A	119.8
C1—O1—Cu	117.55 (17)	C17—N1—C15	105.2 (2)
O2—C1—O1	124.2 (3)	C17—N1—Cu	126.2 (2)
O2—C1—C2	119.4 (2)	C15—N1—Cu	127.76 (19)
O1—C1—C2	116.4 (2)	C17—N2—C16	107.7 (2)
C7—C2—C3	118.2 (3)	C17—N2—H2A	126.5
C7—C2—C1	122.1 (2)	C16—N2—H2A	125.8
C3—C2—C1	119.6 (2)	C20—N3—C18	105.4 (2)
C4—C3—C2	120.6 (3)	C20—N3—Cu	129.63 (18)
C4—C3—H3A	119.7	C18—N3—Cu	124.96 (18)
C2—C3—H3A	119.7	C20—N4—C19	107.5 (2)
C5—C4—C3	120.5 (3)	C20—N4—H19A	126.3
C5—C4—H4A	119.8	C19—N4—H19A	126.3
C3—C4—H4A	119.8	C16—C15—N1	109.4 (3)
C4—C5—C6	119.7 (3)	C16—C15—H15A	125.3
C4—C5—H5A	120.1	N1—C15—H15A	125.3
C6—C5—H5A	120.1	C15—C16—N2	106.5 (3)
C5—C6—C7	120.5 (3)	C15—C16—H16A	126.7
C5—C6—H6A	119.8	N2—C16—H16A	126.7
C7—C6—H6A	119.8	N1—C17—N2	111.2 (3)
C2—C7—C6	120.5 (3)	N1—C17—H17A	124.4
C2—C7—H7A	119.7	N2—C17—H17A	124.4
C6—C7—H7A	119.7	C19—C18—N3	109.5 (3)
C8—O3—Cu	120.30 (16)	C19—C18—H18A	125.2
O4—C8—O3	123.6 (2)	N3—C18—H18A	125.2
O4—C8—C9	119.8 (2)	C18—C19—N4	106.5 (2)
O3—C8—C9	116.6 (2)	C18—C19—H4B	127.1
C14—C9—C10	119.3 (3)	N4—C19—H4B	126.4
C14—C9—C8	120.4 (2)	N3—C20—N4	111.2 (2)
C10—C9—C8	120.3 (2)	N3—C20—H20A	124.4
C11—C10—C9	120.0 (3)	N4—C20—H20A	124.4
C11—C10—H10A	120.0	Cu—O5—H51	106.7
C9—C10—H10A	120.0	Cu—O5—H52	136.7
C10—C11—C12	120.0 (3)	H51—O5—H52	114.7
C10—C11—H11A	120.0	H62—O6—H61	107.1

### Hydrogen-bond geometry (Å, °)

**Table:**

*D*—H···*A*	*D*—H	H···*A*	*D*···*A*	*D*—H···*A*
N2—H2A···O6^i^	0.86	1.88	2.729 (4)	172
N4—H19A···O1^ii^	0.86	2.03	2.886 (3)	177
O5—H51···O2^iii^	0.99	1.96	2.730 (3)	133
O5—H52···O4^iii^	0.97	2.06	2.873 (4)	141
O6—H61···O4^iii^	0.85	1.94	2.729 (4)	155
O6—H62···O3	0.86	1.99	2.827 (3)	165

Symmetry codes: (i) −*x*+1, −*y*+1, −*z*+1; (ii) −*x*+2, *y*+1/2, −*z*+3/2; (iii) *x*, *y*−1, *z*.
